# The Relaxin-3 Receptor, RXFP3, Is a Modulator of Aging-Related Disease

**DOI:** 10.3390/ijms23084387

**Published:** 2022-04-15

**Authors:** Hanne Leysen, Deborah Walter, Lore Clauwaert, Lieselot Hellemans, Jaana van Gastel, Lakshmi Vasudevan, Bronwen Martin, Stuart Maudsley

**Affiliations:** 1 Receptor Biology Laboratory, University of Antwerp, 2610 Wilrijk, Belgium; hanne.leysen@uantwerpen.be (H.L.); deborah.water@uantwerpen.be (D.W.); lore.clauwaert@student.uantwerpen.be (L.C.); lieselot.hellemans@student.uantwerpen.be (L.H.); jaana.vangastel@uantwerpen.be (J.v.G.); 2 SGS Belgium, Intercity Business Park, Generaal De Wittelaan 19-A5, 2800 Mechelen, Belgium; 3 Independent Researcher, 9000 Gent, Belgium; laxvasudevan@gmail.com; 4 Faculty of Pharmaceutical, Biomedical and Veterinary Sciences, University of Antwerp, 2610 Wilrijk, Belgium; bronwen.martin@uantwerpen.be

**Keywords:** relaxin-family peptide receptor 3, aging, G-protein-coupled receptors, DNA, damage, GIT2

## Abstract

During the aging process our body becomes less well equipped to deal with cellular stress, resulting in an increase in unrepaired damage. This causes varying degrees of impaired functionality and an increased risk of mortality. One of the most effective anti-aging strategies involves interventions that combine simultaneous glucometabolic support with augmented DNA damage protection/repair. Thus, it seems prudent to develop therapeutic strategies that target this combinatorial approach. Studies have shown that the ADP-ribosylation factor (ARF) GTPase activating protein GIT2 (GIT2) acts as a keystone protein in the aging process. GIT2 can control both DNA repair and glucose metabolism. Through in vivo co-regulation analyses it was found that GIT2 forms a close coexpression-based relationship with the relaxin-3 receptor (RXFP3). Cellular RXFP3 expression is directly affected by DNA damage and oxidative stress. Overexpression or stimulation of this receptor, by its endogenous ligand relaxin 3 (RLN3), can regulate the DNA damage response and repair processes. Interestingly, RLN3 is an insulin-like peptide and has been shown to control multiple disease processes linked to aging mechanisms, e.g., anxiety, depression, memory dysfunction, appetite, and anti-apoptotic mechanisms. Here we discuss the molecular mechanisms underlying the various roles of RXFP3/RLN3 signaling in aging and age-related disorders.

## 1. Introduction

Aging is arguably one of the most complex molecular biological processes. The majority of eukaryotic organisms undergo the aging process as progressive levels of cellular and tissue damage accumulate across the organism’s lifetime. While bewilderingly complex, aging can be deconstructed as a molecular biological process to reveal a core series of functions that represent a consistent signature that lends itself to potential generic therapeutic interventions. To this end, considerable research has suggested that through the targeting of these key signature features a tractable ability to control the aging process may be engineered. Here we discuss how such a novel target may have been recently identified.

### 1.1. Aging and Aging-Related Disorders

The increase in the world’s elderly population has caused an increased prevalence of aging-related chronic disease conditions, such as neurodegenerative disorders (e.g., Alzheimer’s disease (AD)), cardiovascular disease, arthritis, chronic kidney disease, and Type II Diabetes Mellitus (T2DM) [1]. Aging is a degradative neurometabolic process affecting every organ, and it drives the progression of a multitude of diseases. Aging is a complex multi-factorial process and, while some contributing factors may be unique to each individual, there are many common etiological factors across populations [1,2]. Aging is typified by the accumulation of molecular damage, causing progressive loss of an organism’s optimal function, eventually leading to systemic dysfunction and death [1,3].

Aging and many aging-related disorders involve perturbed energy balance [3]. Regulation of glucose metabolism, via the canonical insulinotropic system, has been shown to be a crucial regulator of the rate of aging [4]. The alteration of energy-controlling organelles and a significant reduction in glucose uptake are a sign of metabolic dysfunction. In times of stress or temporary depletion of glucose supplies, cellular energy metabolism will reflexively shift from glucose to adipose or protein metabolism to guarantee energy production. This metabolic change can cause oxidative stress [5], as the catabolism of these alternative energy sources is less energy efficient and yields lower ATP. The Harman free radical/oxidative stress theory stipulates that physiological iron and other metals in the body cause reactive oxygen species (ROS) accumulation in cells, as a by-product of normal redox reactions. ROS are essentially by-products of a variety of pathways that are involved in aerobic metabolism. The accumulation of oxidative stress constitutes one of the most realistic hypotheses of aging and neurodegenerative disorders [1]. This oxidative stress in turn can cause DNA damage, in the form of double strand breaks (DSBs). While the DNA damage repair (DDR) process functions to repair these DSBs, it is well established that with age, DDR is impaired and can no longer perform this function optimally. This leads to the induction of mutations and/or chromosomal aberration, which in turn can cause cell death, and, in extreme cases, cancer and neurodegenerative disorders [2]. With aging there is a reduced ability to cope with cellular stresses, causing the body to become more prone to a wide variety of pathologies [6]. The central nervous system (CNS), comprised of post-mitotic tissue, is profoundly affected by DDR deficiencies. DDR dysfunction in mature neural tissues is linked to both premature aging and neurodegenerative disorders, such as AD [7].

Aging as a natural pathological process is slowly developing and coordinated by the interaction of multiple signaling systems across several somatic tissues. This complexity makes it a difficult process to therapeutically target. Chadwick et al. [2] demonstrated that such complex systems possess some degree of organization, with some proteins possessing a greater regulatory network function than others. These are the so-called ‘keystones’ (alternatively termed ‘hubs’). Targeting these proteins facilitates regulating these complex disorders, in contrast to controlling the process at every molecular point. One such keystone recently identified is GIT2 (G protein-coupled receptor kinase interacting transcript 2), an ADP-ribosylation factor GTPase-activating protein (Arf-GAP), and a class A G-protein-coupled receptor (GPCR) interacting protein [2,8,9]. GIT2 was identified as an important protein linked to several aspects of the aging process, through latent semantic indexing (LSI). As GIT2 is a potentially important keystone in aging, it might represent a crucial therapeutic target. However, canonical therapeutic targets are receptors, ion channels, kinases, and phosphatases, hence GIT2, being a scaffolding protein, does not represent an effective druggable target [10]. It was recently demonstrated that, in addition to regulating intermediary cell metabolism events such as calcium mobilization, GPCRs can also effectively regulate the expression profiles of multiple signaling proteins via slower signaling modalities outside of the traditional G-protein-dependent functions [11]. This suggests that GPCRs can be used to regulate the expression of specific signaling proteins, to improve therapeutic activity [1,9]. GPCRs are also interesting drug candidates due to their high diversity, targetability, and involvement in nearly every physiological process. Our ongoing research has also demonstrated that the signaling functions of these receptors are far more nuanced than previously conceptualized [2,10]. This signaling complexity facilitates the creation of novel, signal-selective GPCR therapeutics. Previous research, using GIT2 knock-out (KO) mice to investigate expression relationships in the context of metabolic aging, identified a consistently downregulated GPCR, the relaxin-family peptide receptor 3 (RXFP3), in the CNS, pancreas, and liver [10]. This association therefore suggests perhaps that GIT2 may act as a novel aging-specific signaling adaptor for the RXFP3 receptor.

### 1.2. Relaxin-Family Peptide Receptor 3

RXFP3, previously known as GPCR135, was deorphanized through the identification of its endogenous ligand relaxin-3 (RLN3), also known as insulin-like peptide 7 (INSL7). This class A rhodopsin-like receptor, together with its relaxin peptide family and their receptors, is a branch of the insulin superfamily, which consists of insulin and insulin-like growth factor 1 and 2 (IGF1 and -2) [12]. This receptor, originally named the SALPR (somatostatin- and angiotensin-like peptide receptor [13]), is primarily expressed in the CNS [14,15,16]. There are currently four members in the relaxin family of GPCRs, i.e., RXFP1-4. In contrast to RXFP1 and RXFP2, RXFP3 and its closely related family member RXFP4 couple to Gαi, causing inhibition of cAMP production through a pertussis toxin-sensitive mechanism [12]. RXFP3 and RXFP4, also resemble each other in structure, where both are classical type I peptide receptors with short amino (N)-terminal domains, and both are evolutionarily related to somatostatin and angiotensin receptors. In addition, the endogenous ligands for these two receptors are RLN3 and insulin-like peptide 5 (INSL5), respectively, which both play a role in neuroendocrine signaling [17].

While RXFP3 has been classified as a class A rhodopsin-like receptor, it appears that it is not in its entirety a canonical rhodopsin-like GPCR. As detailed by van Gastel et al. [10], RXFP3 does not contain a typical transmembrane domain 3 (TM3) Aspartate-Arginine-Tyrosine ‘DRY’ motif, but instead has a Threonine-Arginine-Tyrosine ‘TRY’ motif. This natural variation of this classical GPCR activation motif may demonstrate altered activation state kinetics, with an augmented level of ligand-independent constitutive activity. Furthermore, the highly conserved ExxxD motif, which is vital for RLN3 binding, has been identified at the second transmembrane domain on the extracellular side [18].

The relaxin peptides are small (approximately 60 amino acids long), and similarly to insulin, share a common two-domain structure with an α- and a β-chain in their mature form [12]. The α-chain appears to be important for receptor–ligand binding affinity, while the β-chain of RLN3 is mainly responsible for binding and activation of RXFP3 [19]. RLN3 is the most recently identified relaxin family peptide, with the presence of the characteristic RxxxRxxI/V relaxin-binding motif found in the β-chain of all relaxin peptides; however, the remainder of the sequence displays low homology with other relaxin-family peptides. RLN3 is the only member of the relaxin-family with a sequence conserved across species [20,21], and this neuropeptide is believed to be the ancestral peptide of the family [20,22]. The RLN3/RXFP3 system demonstrates strong indications of ligand-receptor co-evolution, where nearly all amino acids have been subject to purifying selection for both genes and display a near perfect parallel in both mammals and teleosts [23], both in structure and function [20,23]. Teleosts possess two rln3 paralogs (rln3a and b) and multiple rxfp3-type genes, which are not all orthologous to mammalian RXFP3 [23]. However, it has been shown that intracellular loops 1 and 3 are important in terms of selection, indicating that a large part of selection for these GPCRs concerns downstream receptor signaling and not just selection for ligand binding [23].

**Figure 1:**
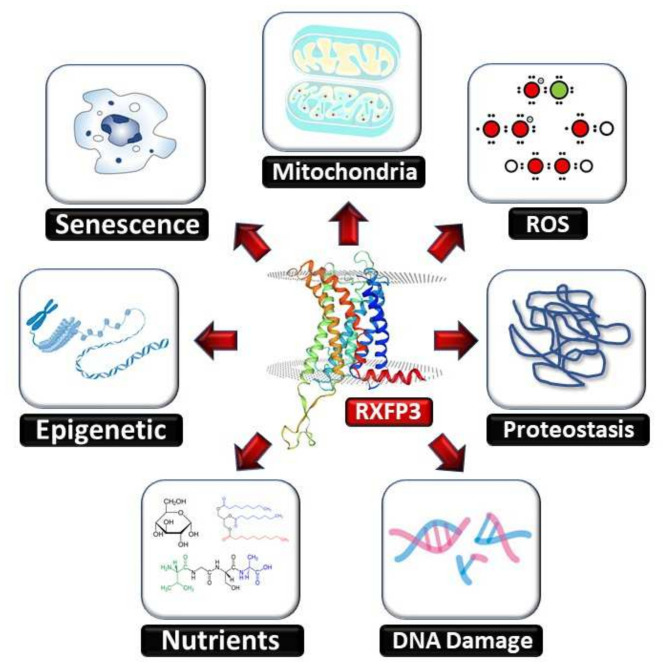
The human RXFP3 receptor functionally intersects with multiple hallmarks of aging. The RXFP3 receptor has been shown by multiple researchers to be associated, at the molecular signaling level, to activities that constitute many of the classical hallmarks of aging. In doing so the RXFP3 potentially represents, in conjunction with its synergistic relationship with the GIT2 signaling adaptor, a novel systems-level therapeutic target for the multidimensional interdiction of the pathological aging process.

Further investigation of the functions of this receptor has uncovered that RXFP3 might play a vital role in several aging-related disorders, as a connection has been found to several hallmarks of aging, such as oxidative stress and DNA damage response [24], similarly to the aging keystone GIT2 [6,7]. In addition, research by other groups has elucidated possible roles for RXFP3 in stress responses [25], anxiety [26], depression [26,27], feeding [15,28,29,30], arousal [28], and alcohol addiction [31]. Given the plethora of possible physiological activities of RXFP3, we will next assess how RXFP3 functionality may intersect with several of the classical hallmark processes involved with the aging process (Figure 1).

## 2. Intersection of RXFP3 Signaling with the Hallmarks of Aging

While the aging process is a complex network of biological processes unique to every individual, there are various common molecular components of the aging process. These components, or so-called ‘hallmarks of aging’, manifest during normal healthy aging, accelerate pathological aging when aggravated and retard normal aging when relieved [32]. López-Otín et al. [32] described nine such hallmarks contributing to the aging process: (1) genomic instability; (2) telomere attrition; (3) epigenetic alterations; (4) loss of proteostasis; (5) deregulated nutrient sensing; (6) mitochondrial dysfunction; (7) cellular senescence; (8) stem cell exhaustion; and (9) altered intercellular communication. Due to the overlap and simultaneous occurrence of these alterations in aging, it is difficult to estimate the relative contribution of each hallmark. In the following section, the involvement of the RXFP3/RLN3 signaling system in multiple processes underpinning several hallmarks of aging will be described.

### 2.1. Metabolic and Mitochondrial Dysfunction

Metabolic syndrome (MetS), which is mainly observed in late-middle aged and older adults, is typified by insulin resistance, and leads to major impairments including adipose lipogenesis, defective glycogen synthesis, and glucose uptake in skeletal muscle. Dysfunction of adipose tissue caused by MetS is widely recognized as a significant hallmark of the aging process [33]. While many older individuals seem to maintain a healthy body mass index (BMI), they are still prone to abdominal obesity, increasing their likelihood of developing MetS [34,35]. Furthermore, aging-related alterations in metabolic pathways and body fat distribution seem to be the active participants in a vicious cycle that is a possible accelerating factor in the aging process, as well as for the onset of many diseases [36]. For cellular metabolic pathways, glucose is the most utilized source of cellular energy and is typically produced from ingested dietary carbohydrates, but it can also be created within the body itself by gluconeogenesis. Glycolysis is the primary mechanism of energy generation in a wide variety of cells and tissues [37]. This mitochondrial process ultimately aims to generate, from glucose metabolism, adenosine trisphosphate (ATP) and reduced nicotinamide adenine dinucleotide (NAD). However, along with this positive effect of mitochondrial energetics, these organelles are also the primary source of ROS, which have been implicated in one of the best-characterized theories of aging, i.e., the oxidative stress theory [38,39]. ROS can cause damage by irreparably affecting the structure of many molecules of the body that are potent controllers of natural aging, e.g., the telomeric regions of DNA [39].

It has been shown that even modest levels of metabolic dysfunction can exert profound effects upon CNS tissues [3,40,41]. This is likely due to different factors, i.e., high energetic requirements of the CNS, combined with a high sensitivity of post-mitotic neuronal tissues to metabolic stress [6,42]. One of the key organs responsible for maintaining an effective interaction between neurological activity and energy balance is the hypothalamus. This small but vital part of the brain is involved in the aging process, as it coordinates both peripheral and central functions associated with neuroendocrine functionality, through the hypothalamic-pituitary-adrenal (HPA) axis [43]. The RXFP3/RLN3 system is highly expressed in different regions involved in the HPA axis, such as the paraventricular nucleus, indicating an involvement in metabolic control [15,44,45,46]. Administration of corticotropin-releasing factor (CRF) has been shown to result in the activation of RLN3 containing neurons in the nucleus incertus, further supporting its functional role in the HPA axis [47]. DeAdder et al. [48] demonstrated that glucose-deprived brain slices display increased cell death and damage, while treatment with RLN3 returned these levels to baseline. Furthermore, blocking the receptor using the RXFP3 antagonist, B1-22R eliminated the effect of RLN3 treatment. Moreover, addition of L-NIL, a NOSII inhibitor, partially eliminated the RLN3 treatment effect. This indicates the involvement of NO synthase in the protective function of the RLN3/RXFP3 system in glucose deprivation [48].

In recent years it has become apparent that mitochondrial dysfunction may be one of the central factors that allow metabolic changes to impact the aging process [49,50]. For the RXFP3 signaling system it is interesting to note that the identified aging keystone factor GIT2 has also been shown to be a potent regulator of mitochondrial functionality [10,51,52]. Given this data, it is not surprising that natural protective mechanisms, e.g., in times of oxidative stress such as in ischemic stroke, that include mitochondrial and respiration support can be affected by relaxin (relaxin-2 (RLN2) and RLN3) peptides [53]. GIT2 has also been shown to be sensitive to ischemic events in multiple tissues [54]. Thus, single nucleotide polymorphism analysis of large patient cohorts identified GIT2 as a marker that confers susceptibility to early-onset MI myocardial infarction), hypertension, or chronic kidney disease.

### 2.2. Oxidative Stress

Oxidative stress refers to an imbalance between the generation of ROS and antioxidants, in favor of ROS, leading to disruption of redox signaling and control and eventually molecular oxidative attack [55]. ROS comprise unstable oxygen radicals (e.g., superoxide radicals and nonradical molecules such as hydrogen peroxide) that, at moderate concentrations, have important intracellular signaling functions, e.g., for the control of nerve transmission and immune regulatory processes. Moreover, low levels of oxidative stress by ROS even appear to be beneficial to organisms. Among others, Doonan et al. demonstrated that it may indeed prolong lifespan in yeast and *C. elegans* [56,57], demonstrating the role of ROS in triggering cell proliferation and survival in response to normal stress conditions and physiological signals [57]. Oxidative exposure of cells occurs naturally as ROS are continually produced during normal aerobic metabolism via the electron transport chain in mitochondria, which is not only a source of ATP, but also ROS [58]. However, ROS are not produced in an unregulated manner, with rates of ROS production typically being extremely low (~0.1 nM H_2_O_2_ formed min^−1^ mg^−1^ mitochondrial protein, ~0.01% of metabolic rate) [56]. Nevertheless, ROS levels may increase in damaged or aged mitochondria which cause accumulation of ROS beyond physiological levels [56]. When their production overwhelms the capacity of antioxidant systems, they can cause irreversible molecular damage to macromolecules (e.g., lipids, proteins, and nucleic acids) and accumulated cell disruption, affecting multiple cellular functions, which over time is associated with cellular senescence and aging [6,58].

Van Gastel et al. [24] identified superoxide dismutase 1 (SOD1), sirtuin 1 (SIRT1), Ras GTPase-activating, and peroxiredoxin 6 (PRDX6) among the proteins functionally interacting with RXFP3, indicating a role in oxidative stress responsiveness. Loss of PRDX6 expression has previously been observed in aging cells and was shown to increase ROS production [59]. Furthermore, slight overexpression of RXFP3 resulted in an increased expression of PRDX6, indicating a synergistic role in the response to aging and oxidative stress [24]. Similar to PRDX6, SOD1 is also upregulated with RXFP3 overexpression [24]. Deletion of SOD1 in yeast and mouse models leads to increased oxidative stress and DNA damage. Elevated oxidative stressors, such as hydrogen peroxide, regulates the nuclear localization of SOD1. This process is associated with the Ataxia-Telangiectasia-mutated (ATM)/mec1 serine/threonine protein kinase (Mec1) regulation of gene expression to prevent oxidative stress-related DNA damage [60]. Similar to SOD1, the SIRT1/FoxO axis is important for the regulation of the response to metabolic and oxidative stress through the overexpression of antioxidants [61]. Recent evidence has also demonstrated that relaxin-3, acting via RXFP3, possesses the capacity to attenuate oxidative damage induced by glucose deprivation in cultured brain slices, through the manipulation of the nitric oxide generation system [48]. The specificity of this effect of relaxin-3 at the RXFP3 was demonstrated by a selective inhibition through the action of the RXFP3 antagonist, B1-22R [48]. Taken together it seems that RXFP3-associated signaling complexes (often referred to as receptorsomes [10,24]) could act as a sensor for oxidative stress and regulate the cellular response to it.

### 2.3. DNA Damage

The amino acid sequence of RXFP3 displays multiple phosphorylation sites for kinases involved in DDR (i.e., ATM/PRKDC at serine 269 and 360: https://scansite4.mit.edu/, accessed on 12 April 2022). This could explain its association with GIT2 in aging and neurodegeneration. As discussed, DNA damage is one of the hallmarks of the aging process [32]. It has been demonstrated that many advanced aging disorders are linked to mutations in DDR proteins, e.g., a mutation occurs in ATM causing Ataxia-Telangiectasia (AT) [62]. ATM plays a central role in the maintenance of genome stability and phosphorylates proteins involved in the canonical DDR process. The phosphorylation preferentially takes place on serine (S) or threonine (T) residues preceded by glutamine (Q), the so-called SQ/TQ motifs [63]. This is required for normal DNA damage repair [63]. Interestingly, some researchers have demonstrated that ATM protein kinase is a sensor for ROS in human cells, concluding that ATM can be directly activated by oxidation [64]. RXFP3 contains two SQ motifs, which strongly suggests the potential involvement of RXFP3 as a sensor for oxidative stress, leading to aging. RXFP3 also contains a phosphorylation site for PRKDC. PRKDC binds to SxQ motifs, which in the case of RXFP3 x is a leucine (L), found in the receptor sequence in extracellular loop 2. In classical class A GPCRs these ATM and PRKDC sites would be located in the intracellular domain, whereas in RXFP3 they are located in the extracellular loops. However, it is highly likely that in this case the sites are still accessible to intracellular ATM/PRKDC. Recently it has been hypothesized that GPCRs can be inserted inside-out in intracellular membranes such as the nucleus, endoplasmic reticulum, or mitochondria [65,66,67]. This would mean that the extracellular loops are accessible by intracellular ATM/PRKDC. Moreover, it has also been shown through surface accessibility topological predictions that these three sites are likely to be accessible to soluble hydrophilic factors. It has also been demonstrated that the majority of GPCRs are held in intracellular vesicles as a receptor reserve for plasma membrane recycling [68], and the TRY motif likely increases the amount of intracellular retained receptors [69].

Besides the phosphorylation sites for ATM and PRKDC, it was found that activation of RXFP3, through its endogenous ligand RLN3, increased PRKDC phosphorylation while resulting in a decrease of histone H2AX (H2AX) and breast cancer type 1 susceptibility protein (BRCA1) phosphorylation [24]. H2AX phosphorylation is one of the first molecular indicators of DNA damage that can then be repaired through the activity of BRCA1. Co-immunoprecipitation, using selective affinity purification of an N-terminally haemagglutinin-tagged RXFP3, also indicated interaction of RXFP3 and activated PRKDC, highlighting the importance of RXFP3 in DNA damage repair through PRKDC [24].

### 2.4. Epigenetic Alterations

Epigenetic alterations to nucleic acids are a component of the normal cellular signaling landscape. Alterations to patterns of epigenetic profiles across the aging process are potentially one of the key factors for controlling individual healthy aging trajectories [70,71,72]. As aging is linked with altered epigenetic mechanisms of gene regulation, such as DNA methylation, histone modification and chromatin remodeling, and non-coding RNAs, the potential therapeutic control of these mechanisms is a potentially effective strategy for interdicting the generation of pathological aging phenotypes. It has been shown that the methylation status of RXFP3 can be associated with aging-related changes in several cancers, including endometrial and cervical malignancies [73,74,75]. Alterations to the methylation status has also been shown for several other receptors with respect to these specific cancers, e.g., orexin-2 receptor [76], C-X-C chemokine receptor type 4 [77], and the P2X purinoceptor 7 [78]. It is interesting to note, however, that with respect to the role of RXFP3 in the aging process, Huang et al. [79] identified coordinated changes in RXFP3 epigenetic regulation along with CIDEA (cell death activator CIDEA), which has also been implicated in metabolic pro-aging molecular signaling activities [80].

### 2.5. Nutrient Sensing

The complex and intricate aging process is implicitly associated with the glucometabolic system. Indeed, many of the first aging-regulating genes discovered in species such as *C. elegans* were nearly all associated with the insulinotropic system [81,82]. Given this, it is also interesting to note that the metabolic underpinning of nearly all diseases is now apparent, demonstrating the importance of therapeutic intervention in these systems [83,84,85,86,87,88,89,90,91,92]. From an intervention standpoint, simple lifestyle modifications have subsequently demonstrated that caloric restriction (CR) can be effective in controlling the glucometabolic system to attenuate the incidence and magnitude of aging-related disease [93,94,95,96,97,98,99]. Thus, it is clear that the ability of cells and tissues to sense fuel sources for energy metabolism is critical for the maintenance of homeostasis across lifespan [100]. The main components of signaling pathways sensitive to the changes in the nutrient availability include insulin, TOR (target of rapamycin), AMPK (5′-AMP-activated protein kinase), and the sweet-taste receptor system [86,101,102]. Impairments of these signaling pathways can trigger various metabolic disorders. Thus, disrupted functioning of AMPK can reduce the stress resistance capacity of cells as well as engendering the development of insulin resistance [103,104]. Activation or suppression of metabolic sensors might increase lifespan in various organisms and improve aging-related indicators in humans [105,106,107].

While a considerable amount of nutrient sensing is controlled by factors directly linked to the insulinotropic system, there are multiple other systems (including GPCRs) that also regulate the functionality of this longevity regulating paradigm. In this light, it is interesting to note that RLN3 is an insulin-like peptide that has also been shown to be a controller of nutrient sensing and catabolic metabolism [108,109,110]. Demonstrating a further nuance of the positioning of the RXFP3 system, it has also been shown that regulation of food intake is also associated with psychosocial changes, suggesting that RXFP3 can act as a nexus between generic stress responses and cellular protection mechanisms to combat the deleterious effects of nutrient deprivation [29]. To further investigate this, it would be interesting to investigate such a proposal through the implementation of RXFP3 antagonist introduction or tissue-selective RXFP3 expression attenuation or silencing with short hairpin RNA or CRISPR/Cas9 approaches.

### 2.6. Cell Senescence

Aging-related diseases are caused by the progressive degradation of the integrity of communication systems within and between organs. This process is associated with a decreased efficiency of receptor signaling systems and an increasing inability to cope with stress, leading to apoptosis and cellular senescence [111,112,113]. Cellular senescence is a natural process during embryonic development but more recently it has been shown to also be involved in the development of aging-related disorders and is now considered to be one of the major hallmarks of aging. Advances in the molecular understanding of GPCR signaling complexity have expanded their therapeutic capacity tremendously [114,115,116,117,118]. Thus, emerging data now suggest the involvement of GPCRs and their physically associating adaptor proteins in the development of cellular senescence [119,120,121]. With the proven efficacy of therapeutic GPCR targeting, it is reasonable to now consider GPCRs as potential platforms for controlling cellular senescence and aging-related disorders. RXFP3 has been functionally associated with the senescence process in several studies. Recently Anckaerts et al. [122] demonstrated that interventions that induced premature brain aging and senescence (without excessive cell loss) in the context of AD resulted in significant diminutions in both RXFP3 and GIT2 expression in the retrosplenial cortex. Senescent cellular programs, especially in the aging context, are often induced by the overburdening of cells with oxidative stress. Several studies have linked this deleterious process to the significant alteration of RXFP3 expression levels [24,123] as well as the ROS regulating factor PRDX6 [24,124]. PRDX6 has subsequently been shown to be a crucial integrator of aging-associated cellular senescence programs [59].

### 2.7. Proteostasis/Fibrosis

Maintaining cellular protein homeostasis, or proteostasis, requires the well-coordinated control of protein synthesis, folding, conformational integrity, and ultimately degradation. Proteostasis activities coordinate these diverse processes across the lifespan of all organisms [125]. The proteostatic regulatory network ensures that cells have the proteins they need, while minimizing misfolding or aggregation events that are hallmarks of aging-associated proteinopathies, such as Alzheimer’s, Parkinson’s, and Huntington’s disease [126,127,128,129,130]. It is now clear that the capacity of cells to maintain proteostasis undergoes a decline during aging, rendering the organism susceptible to these pathologies. One of the most common pathological sequalae of altered proteostasis is the dysfunction of basement complexes (an extracellular matrix network of glycoproteins and proteoglycans) that can cause fibrosis in multiple tissues across the lifespan [41]. Given our previously demonstrated evidence regarding the potential anti-aging activity of RXFP3 [24], it is not surprising that components of the RXFP3/RLN3 system have been shown to have antifibrotic activity. For example, Hossain et al. [131] showed that RLN3, albeit acting via the RXFP1 receptor, could decrease collagen expression in a murine cardiomyopathy model. It has also been shown that RLN3 treatment of cardiac fibroblasts inhibited ROS- and inflammasome-mediated collagen synthesis under high glucose conditions [132]. In addition to this, in the context of cultured cardiac fibroblasts, exposure of these cells to hyperglycemic conditions (that predisposes to fibrosis) causes an elevation in mRNA levels of RXFP3 [133]. Based on this data, it is clear that RLN3/RXFP3 signaling could represent a novel therapeutic avenue for diabetic cardiomyopathy [133].

## 3. RXFP3 in Aging-Related Disorders

As discussed, the aging process is largely driven by glucometabolic dysfunction. Hence, conditions such as T2DM or MetS are potent triggers of multiple forms of aging-related disease. Therefore, it is important to consider that the mechanisms through which glucose metabolism becomes dysregulated over time require more in-depth investigation. While the insulinotropic system is the primary mechanism for controlling glucose uptake and use, it has been shown in recent years that there are many other receptor systems (especially GPCRs) that also potently regulate glucose metabolism [86,128,134,135,136,137]. Here, we propose that the RXFP3 system, especially when it is actively interacting with GIT2, also forms part of this glucometabolic family [2,5,24,133]. Even though there has been a significant focus on investigating the role of glucose metabolism in the aging process (potentially via the profound link with mitochondrial support in aging), there is also the strong impact of the adipose tissue system in this paradigm [138,139,140]. Reinforcing the potential importance of RXFP3 in the aging process, it has been demonstrated that RLN3 can play an important role in adipogenesis and maturation [141]. This functionality may not be entirely unexpected as RXFP3 appears likely to be a manager of energy metabolism in times of aging-associated metabolic disruption [10]. Hence, RXFP3 has been associated with the functionality of energy metabolic systems involving dietary-related weight gain, insulinotropic functions, and adipogenic activities that are strong players in the aging process [3,10,29,138,142,143,144]. In the next sections we will highlight the contributions and activities of RLN3/RXFP3 signaling in aging-related disease. These insights strengthen the concept that the RXFP3-GIT2 signaling system may represent a novel signaling relationship system that can be developed for novel and effective anti-aging therapeutics.

### 3.1. Alzheimer’s Disease

It is now well appreciated that many classical central nervous system neurodegenerative disorders, such as Alzheimer’s, Parkinson’s, and Huntington’s disease, share many common etiological features with perhaps the primary one being metabolic dysfunction [126,128,145,146]. With respect to AD, it has been shown that RXFP3 levels are significantly altered in the neocortex of depressed Alzheimer’s patients [147]. Alzheimer’s disease is primarily represented as a dysfunctional capacity for short-term memory formation and then, at a later stage, a dysfunction in long-term memory recollection. It is relevant to note that depletion of RXFP3 levels in the brain have been associated with long-term memory regulation in adult mice [27]. In addition to long-term memory recall, RXFP3 functionality has also been associated with spatial memory formation [148,149].

### 3.2. Anxiety and Post-Traumatic Stress Disorder

Anxiety, along with associated disorders such as post-traumatic stress disorder (PTSD), have in recent years been strongly associated with premature aging conditions [150,151,152,153]. With respect to the impact of RXFP3 upon anxiety-related disorders, it has been shown that specific central stimulation generates an anxiolytic effect in model organisms [154]. While acute effects of RXFP3 stimulation can generate anxiolytic effects, it has recently been shown that chronic localized RXFP3 stimulation instead can actually promote anxiety behavior [26]. Thus, it appears that the anxiety-related activities of RXFP3 may be highly context specific in experimental animal models [155]. Such a phenomena therefore entails a more detailed view in human patients of this specific situation and therefore RXFP3-based interventions could potentially be therapeutic targets for certain forms of anxiogenic activity.

It is interesting to note that the RXFP3-GIT2 signaling axis seems to be a priority signaling system for central anxiety/stress conditions, as not only is GIT2 involved in anxiety-related behavior directly, it is also a potent regulator of the glucose metabolic system that intertwines with anxiety-related conditions [156,157]. Moreover, it has been shown that both RXFP3 and GIT2 are highly expressed in the amygdala [123,158,159]. There is also evidence to suggest that through common activities related to stress responses RXFP3 and GIT2 together may contribute in a coordinated manner to interconnect anxiety behaviors [158] and stress responses such as hyperphagia or binge-eating [160,161,162]. This anxiety-related condition will likely then feed into the generation of metabolic dysfunction via metabolic or diabetic syndromes. While impulsive behavioral responses in response to stress are seen with food, there is also considerable evidence that this stress-induced activity also includes augmented alcohol seeking activity [163].

### 3.3. Schizophrenia

Our recent work has begun to provide evidence for the aging-related control of schizophrenia and schizophrenia-related conditions [164,165,166]. Relaxin ligands, as well as RXFP3 itself, have been suggested by some researchers to be implicated in schizophrenia-related conditions [167,168]. Again, demonstrating the metabolic basis of aging, it has been shown that relaxin-3, RXFP3, and RXFP4 polymorphisms have been linked to metabolic disruptions in patients treated with anti-psychotic medications [169]. Schizophrenia and other affective conditions are typified by periods of mania and heightened activity states, and it has been demonstrated that cognitive arousal states can also be strongly affected by RXFP3 activity in experimental animal models [144]. In regard to the potential for a specific RXFP3-GIT2 signaling axis in the aging process, it is interesting to note that epigenetic modifications (hypermethylation) of GIT2 have recently been shown by the creation of schizophrenic differential methylation networks (SDMNs) from schizophrenia patient data [170]. The specific effects of this modification of GIT2 in this paradigm has, however, yet to be shown [164].

### 3.4. Obesity and Metabolic Dysfunction

Multiple experimental animal and longitudinal studies have demonstrated that diet-induced obesity promotes pro-aging phenotypes [84,97,171,172]. A considerable component of the obesity-based drive of aging likely results in alterations in insulin sensitivity as well as the drive towards alternative sources of energy, such as lipid- or protein-mediated metabolism that can incur a greater level of oxidative stress [173,174,175].

RXFP3 expression and activity has been shown to be closely associated with both eating behavior alterations [161,176] as well as the physiological responses to augmented food intake [160,177,178,179]. In many of these experiments it has been noted that the role of RXFP3 in these scenarios is more pronounced in females compared to males [177]. Concordant with this, it has been shown that female RXFP3 knock-out mice present with more heightened anxiety behavior than male RXFP3 knock-out mice in assessments of anxiety, such as the elevated plus maze. Hence, male RXFP3 knock-out mice spent more time in the open arms of the maze indicating their lower levels of anxiety than their female RXFP3 knock-out counterparts [144]. Experimental animals fed a high fat/glucose diet (a common mechanism to accelerate metabolic aging) displayed significant alterations in the CNS expression of RLN3 and RXFP3 [178]. These diet-induced obese (DIO) male rats displayed significantly higher levels of RLN3 expression compared to control diet ad libitum fed animals. This increased expression of RLN3 in DIO rats likely engenders the hyperphagic condition found in this experimental cohort. This study found that during a metabolic challenge of refeeding after food deprivation, the DIO rats only exhibited an increased expression of RXFP3 receptors in brain regions involved in food intake regulation [178]. With respect to the links between the RLN3/RXFP3 system and human obesity it has been shown that RLN3 genetic polymorphisms are significantly associated with traits including obesity, hypercholesterolemia, and diabetes [169]. The intersection of RLN3/RXFP3 signaling between stress-responsive binge eating and this greater role of RXFP3 in predisposition to obesity demonstrates the importance of this system in the control of glucometabolic dysfunction in the aging context. Given these associations, considerable activity has since focused on the development of RLN3-based interventions for obesity paradigms [162,179,180].

### 3.5. Ischemic Stroke

Aging is considered to be one of the strongest independent risk factors for ischemic stroke-based injuries [181,182]. Hence, almost three-quarters of all strokes occur in people aged ≥65 years. Ischemic damage is cellular destruction associated with altered nutrient or oxygen support–resulting in energy deprivation and ROS-based damage. A recent study reported that relaxin peptides can protect tissues from ischemic damage. Using a rat stroke model, it was demonstrated that RXFP3 activation (using RLN2 and RLN3) reduced the extent of cellular/tissue damage induced by the application of vascular ligation [53]. In this study it was reported that the ability to reduce the size of infarcts induced by transient middle cerebral artery occlusions was primarily mediated by selective activation of RXFP3. In addition to this, RXFP3 stimulation also demonstrated the capacity to reduce the damaging effects of oxygen and glucose deprivation in cellulo-cultured primary astrocytes.

### 3.6. Reproductive Aging

The control of reproductive behavior is tightly linked to the functional cellular/tissue mechanisms associated with energy metabolism and food availability [59,98,122,183,184,185]. As reproductive behavior and physiology are tightly controlled at certain points in the lifespan, it is not surprising that the broader relaxin system likely intersects with this aging–reproduction nexus. The role of relaxin in the reproductive process is one of the best studied aspects of its molecular biology [186,187,188]. In a recent study that investigated the effects of premature defects in the female reproductive system (i.e., ovariectomy) it was found that in areas of the brain that demonstrated a dysfunctional network connectivity, there was a significant alteration in the levels of both RXFP3 expression and its potential preferred partner, GIT2 [122]. Thus, it is likely that this receptor system [10] can also form a functional bridge between the aging process and the reproductive system.

### 3.7. Alcohol Abuse

Alcohol use disorders are a leading cause of preventable deaths worldwide. Sobering patients often experience alcohol use relapses in times of physical and psychosocial stress. Both RLN3 and RXFP3 have been shown to modulate stress-induced relapse to alcohol seeking in rats. The amygdala is one of the most crucial areas of the CNS that controls this pathobiology. The central nucleus of the amygdala (CeA) in the rat receives a RLN3 innervation and possesses considerable levels of RXFP3 expression. In addition to this, the CeA receives considerable input from corticotropin releasing factor (CRF) neurons demonstrating a functional intersection between stress and this activity of the RLN3/RXFP3 system. In this specific scenario it is thought that CeL (lateral CeA) CRF neurons provide both local inhibitory GABA and excitatory CRF signals to the CeA neurons [189].

As discussed previously, alcohol seeking behavior can be a major component of PTSD/anxiety phenotypes [190]. Recent research has also indicated that the response to alcohol intake is also affected by the age of the afflicted individual [191]. As we have contended that these stress-related conditions are potentially driven by metabolic disruption, it is unsurprising that recent research has started to propose that alcoholism behavior is also linked to pathological aging [192,193,194]. Recent evidence has indicated that alcoholism can even lead to Alzheimer-like conditions that have a strong neuroinflammatory component [195]. Excessive and inappropriate alcohol abuse results in the generation of multiple co-morbidities, including neurodegenerative atrophy, dysfunctional immune responses, and accelerated or premature aging [194,196]. One of the better studied functions of RXFP3 has been its regulatory capacity in alcohol seeking behavior [26,163]. In contrast to the RXFP3-based actions on feeding behavior [177], it has been shown that only male RLN3 knock-out animals showed an increase in alcohol preference [197]. Thus, it is possible that RXFP3-based therapeutics could be a potential future target for the treatment of alcoholism.

## 4. Conclusions

Aging is one of the largest risk factors for nearly every type of major mortality-causing disease in the world today. Therefore, tractable mechanisms for controlling this highly complex process are urgently required as a molecular target for intervention. The therapeutic interdiction of the aging process is currently one of the most studied therapeutic areas. Interventions in the aging process often fall into either lifespan extension strategies or damage/disease reduction strategies. While lifespan extension is an interesting goal [198] interventions that seek to reduce the accumulation rates of damage [199,200] may be more likely to impact medicine more immediately. With respect to lifespan extension, one of the most studied current modes of intervention is the cellular rejuvenation process. In this context it is often proposed that through selective genetic modulation of longevity regulating factors a reversal of aging-related damage can be achieved [198,201]. Several prominent reports have indeed suggested that reversal of aging damage can occur, e.g., in vivo ectopic expression of three (Oct4, Sox2, Klf4) of the four Yamanaka reprogramming factors [202] was able to promote axon regeneration after previous eye injury [203] and also stem multiple aspects of aging-related disease such as renal failure, cardiomyopathies, and diabetic conditions [201]. While generating dramatic results these interventions are still at the experimental animal phase and will be unlikely to transition to the human stage soon.

Lifestyle (e.g., exercise) and dietary interventions (e.g., caloric restriction) have been shown to be effective in slowing down the molecular aging process [95,98,99,204,205] in controlled experimental conditions, however the adherence of human patients to these is often poor and difficult to maintain for long periods of time [206]. Exercise and caloric restriction are proposed to exert beneficial effects through natural augmentations of cytoprotective systems through the introduction of a mild stress. An alternative method to induce this stress is via a controlled exposure to other stressors such as heat, cold, or mild irradiation [207,208,209]. While interventions such as caloric restriction and exercise are still problematical for adherence, these even more drastic mild stressors are much less likely to be accepted by a clinical audience.

**Figure 2:**
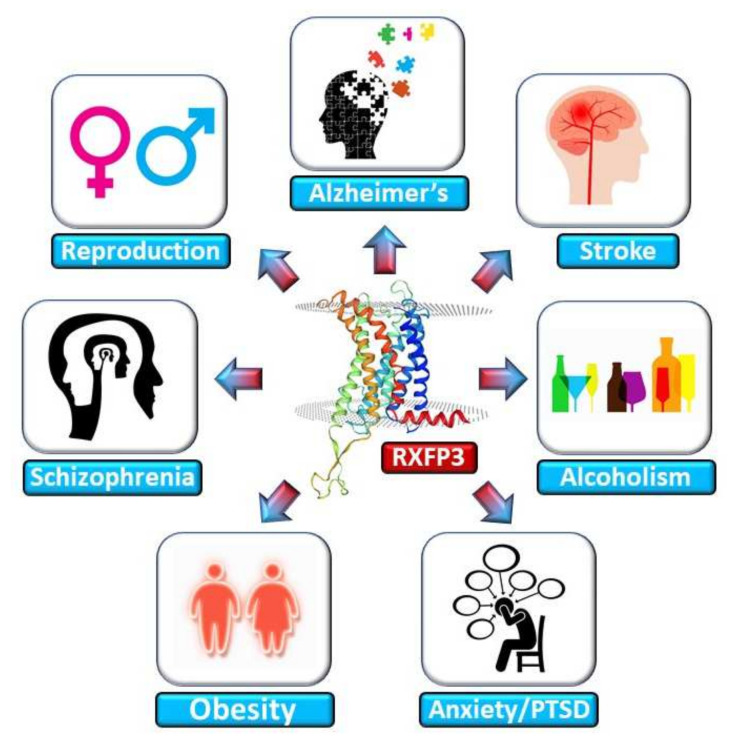
The human RXFP3 receptor is involved in multiple disorders associated with dysfunctional aging. Alterations in the activity and expression of the human RXFP3 receptor have been shown by multiple research teams to play a pivotal role in the disease processes depicted. The participation of RXFP3 in these disorders indicates a role for perturbed natural aging signaling mechanisms in these conditions. Hence, it is likely that further investigation of the diverse signaling capacity of RXFP3 may help generate novel therapeutics for these conditions that work via altering the rate of aging in these disorders.

In addition to genetic or lifestyle interventions the use of polypharmacological natural compounds, e.g., quercetin or resveratrol [171,210], has gained significant interest in recent years but has often stalled following the transition from experimental conditions to more clinical settings [211,212]. It is likely that the complex polypharmacological actions of these natural agents could be the issue with respect to this transition, as many of these effects may be specific to smaller groups of patient populations and may also be highly influenced by diet and compound metabolism variation. The concept of tackling complex disorders, e.g., pathological aging, in a polypharmacological manner may indeed be a good strategy as many systems may need remediation. The natural compounds may indeed exert systemic beneficial effects, but as that could be through a variety of molecular targets a coherent response profile in patients may be hard to obtain. A pragmatic approach may be to identify natural receptor signaling systems that present an ability to interdict pathological aging at a systemic level, e.g., the RXFP3/RLN3 system, especially when combining with the GIT2 signaling paradigm. Our research, as well as that of others, has identified RXFP3 as a potential crucial factor in controlling both the classical hallmarks of molecular aging and the etiological process of multiple forms of aging-associated disease (Figure 2). Moreover, our research outlined here also indicates that there are multiple points of intersection between the RXFP3 signaling paradigm and molecular signaling mechanisms of aging-associated disease. We have previously shown that RXFP3 possesses a strong functional relationship with the aging keystone, GIT2. Thus, this synergistic relationship presents as a completely novel therapeutic mode for attenuating aging pathology in a multidimensional manner. In this intervention avenue there is a strong systemic anti-aging component combined with the capacity to generate more selective and specific molecular intervention, compared to naturally occurring compounds. Hence, this approach is a form of engineered polypharmacology. To further advance this research it would be interesting to generate signal-selective compounds that target the RXFP3 in a manner that specifically stimulate the RXFP3 to generate GIT2-dependent signaling outputs. This agent could then be introduced to ex vivo or in vivo experimental paradigms to demonstrate a capacity of such agents to ameliorate aging-associated damage and also aging-induced disease phenotypes.
